# Aseptic vs. Antiseptic Surgery*A paper read before the Medical Association of Georgia, at Brunswick, Ga., Thursday, April 17, 1890, at 10 o’clock.

**Published:** 1890-06

**Authors:** Samuel C. Benedict

**Affiliations:** Athens, Ga.


### Southern Medical Record.

A MONTHLY JOURNAL OF MEDICINE AND SURGERY.

VOL. XX. ATLANTA, GA., JUNE, 1890. NO. 6.

### Original Articles.

### ASEPTIC VS. ANTISEPTIC SURGERY.*

SAMUEL C. BENEDICT, M. D., ATHENS, GA.

To you, gentlemen of the Medical Profession of Georgia, who graduated prior to the year 1880, and who have been actively engaged in your work since that time, with no opportunities for keeping up with the rapid advances made in the past decade, other than by books and periodicals, do I chiefly address myself upon this subject, yet so recently in its infancy, and yet so fully and so often discussed. Like yourselves, I have had no way of learning how to do surgery as now performed other than by practical experience, prefaced by many failures, but so wonderful are the results when the teachings of Lister, Gerster, Wyeth and others are faithfully carried out and so fascinating does surgical work become to any one who has the least desire to practice it, I have thought that it might be an acceptable topic for discussion to many of you gentlemen, who, like myself a few years ago, are striving to keep up with the advancement in modern surgical procedures, solely by working out the problem from your own practical experience.

At first the temptation is strong to rush at once to the excessive use, the profuse waste of *germicidal* agents, under the

*A paper read before the Medical Association of Georgia, at Brunswick, Ga., Thursday, April 17, 1890, at 10 o'clock.

belief that by their aid alone we could get the results we so longed for, that is, union of divided and separated portions of the living body in a very few days, with none, or at least, very few changes of dressing and no suppuration. In some cases such a procedure may be followed by the desired results ; antiseptic precautions may have been all that was intentionally used, but unless with the antiseptic agencies we carried out in its fulness of detail, the idea of perfect asepsis, *perfect cleanliness*, we can never say with any degree of assurance that we would not have the same old story of suppuration, granulation and repair, or putrefaction, pyæmia and death. If we could do away with germicides, would it be possible for us to have aseptic surgery? Is it not absolutely necessary that we make use of the principles of antisepsis more than asepsis?

Gerster in his work on aseptic and antiseptic surgery divides his operative surgery in healthy non-suppurating tissues into aseptic, and his treatment of affections due to phlegmon, tubercle, syphilis, etc., into antiseptic work, and yet in all his operative work he uses bicloride solutions, carbolic acid and iodoform as germicides. It may be true that this insures cleanliness as regards the micro-organisms, but without the presence of bacteria, if such a condition was possible, could we secure aseptic results without the use of germicides? To my mind, as I have reasoned it out, after some years of practical experience, and the reasoning that necessarily follows such experience, asepsis and antisepsis in their practical application, are one and the same, and without both of them, as taught, we cannot secure the results we wish. A few surgeons get brilliant results from what they term asepsis, scoffing at those operators who insist upon the use of germicides, and yet, do not they invariably use boiling water for their instruments, water that has been boiled for all uses about the wound, and generally some chemical sterilizer in their solutions? I cannot satisfy myself that we dare to eliminate the bichloride solutions from our operative work, even though that be upon the abdominal cavity. We may boil our instruments, we may flush our wounds with water that has been boiled, and as near the boiling point as we dare to use it; and yet some stray cocci are so liable to be left in the recesses and folds of the tissues, no matter how thoroughly we may try to "clean" the wound, that often we will find in a few days that our dressings are absorbing pus, and that we have failed in attaining the beautiful results we had hoped for.

The title of this paper has been worded as it is, "Aseptic vs. Antiseptic Surgery." for the express purpose of defining, if there be any such distinction, the difference to be understood between the words "asepsis," and "antisepsis." It may seem to you, who have given the subject much thought, to be simply a refinement of words, but may they not be so used as to convey separate and distinct ideas? Gerster in his work uses them in conjunction, but for the purpose of this article and for discussion, I prefer to contrast the two words as applied to surgery. Now what are we to understand by "aseptic" surgery, strictly? Surely surgery *without* sepsis, *without* suppuration, *without* tissue death, however this may be brought about, whether accomplished by the use of antiseptics or not.

It is now known that it requires in some quantity the presence of living germs in the wound to produce sufficient impression as to result in injury to the tissue-cells, and the evolution of ptomaines. May we not by mechanical means alone free the wounds of such germs without destroying them? By this I mean, that without supplying germicides directly to the cut surfaces, may we not have an aseptic wound? I do most certainly answer this in the affirmative, and I believe that what Tait, and our own Battey, and others have accomplished, in abdominal surgery, by the use of sterilized hands, instruments and solutions, may also be obtained in other operations, if the same attention be given to details, and the same dexterity and rapidity in operating be used. This I shonld call aseptic surgery, no matter whether or not we used the antiseptics of heat at 212 degrees F. or such chemical sterilizers as mercury bichloride for external cleanliness. In an editorial in the Medical Record for Nov. 17th, 1888, is a reference to a paper by Dr. E. Saenger in the Medical Zeitung of 1888, in which he says upon the subject, "No doubt strong antimycotic solutions such as sublimate and carbolic acid destroy the staphyloccus aureus, but the weaker solutions do not do this." The disinfection of a living tissue he considers impossible, for either the tissue is free from germs, in which case there is no need for an antimycotic, or it is infiltrated with pathogenic fungi, in which case it is impossible to destroy the fungi without, at the same time, seriously injuring the tissue cells. Disinfection, he says, answers admirably on dead objects, but in case of living organisms more importance must be attached to vital energy. * * * "By all means," he adds, "let dead objects such as instruments, the operating room, etc., be thoroughly disinfected, but after the first incision, no antimycotic whatever should be used." Again, on p. 258 vol. 1, Ref. Handbook of Med. Sciences is this statement: "Aseptic surgery includes a series of measures calculated to absolutely exclude every living germ from lodgment on the wound surfaces, or in the dressings. To this end there is but one known method, and that is the strictest form of Listerism. * * * Let us remember that their strict and conscientious observance constitutes our positively aseptic surgery."

Unquestionably all sepsis is due to the work of germs coming from without the body, that is, through the medium of the air or water, or substances containing them and brought in contact with freshly divided tissues. The air in the neighborhood of slaughter houses, post mortem rooms and of crowded habitations is necessarily more nearly saturated with bacteria than the air of purer localities, such as the country. At first, when Listerism was in its infancy, the spray was used to disinfect the surrounding atmosphere, but that has now been abandoned, since it has been found that equally aseptic wounds could be made by careful attention to cleanliness of the skin surface around the seat of operation, and of the hands and instruments of the operator, combined with either a mechanical dislodgment of the germs by water, sterilized by heat, or by the addition of the sublimate.

The term "Aseptic Surgery," I would apply then to operative surgery upon the tissues which we know, prior to our first incision, were free from all evidences of suppuration, and in which, until that incision was made, there was no possibility that there lay any stray cocci, even though such cleanliness be the result of the use of antiseptics externally.

Water that has been boiled, and is used near the boiling point, I do not consier an antiseptic, because a temperature less than 212 degrees F. will not destroy the life of bacteria. Such water is simply sterilized, and acts mechanically only when it frees a wound of germs. The actual cautery is antiseptic, and may be used to secure an aseptic wound. Operations made without the use of chemical sterilizers to the wound or in the dressings, I would class as aseptic, and under this head would be included the abdominal work of Mr. Tait, and indeed, that of most operators, as now made for laparotomies. That this aseptic mode of operating could be extended to the operations upon other portions of the body, and with equally good results, I believe it possible, but exceedingly improbable, from the fact that the peritoneal cavity affords too poor a pabulum for bacteria, the absorptive power being so great, and because in most all other operations of importance, the area of divided tissues is greater.

In contradistinction to *aseptic*, we have *antiseptic* surgery; surgery directed *against* a septic condition which we know to be present, as operations upon abscesses, pus cavities, necrosed bone, etc. Here we know that we have to dislodge bacteria already within the tissues upon which we are to operate, and here simple sterilized solutions and dressings will not answer. We must destroy by chemical germicides the micro-organisms which are present if we would have an aseptic wound. In aseptic surgery, as I have attempted to define the term, we endeavor to prevent the introduction of bacteria; in antiseptic surgery we dislodge and destroy the germs already introduced, and which are destroying the tissue cells, and producing the constitutional effect of the absorption of ptomaines, the result of such destruction. *Asepsis* is then keeping a tissue *without* septic infection; *antisepsis*, operating *against* a sepsis already present.

This definition leads us to the distinction which Dr. Gerster makes in his work, and which, to my mind, seems an exact and admirable definition. Acute surgery, such as required for trauma and operative surgery upon non-suppurating parts he classifies as *aseptic;* chronic surgery, such as needed for caries and necrosis either of soft parts, or of bone, he call *antiseptic* surgery.

So much for the purposes of this discussion. In practical application of the terms, I believe it is far preferable that we should discard the term "*aseptic*" surgery and call all work where we use germicides with a view to thorough sterilization as "antiseptic" surgery, for in *both* forms of work, whether directed to prevent the introductions of germs into freshly made wounds, or in removing them from parts already septic, our efforts are directed *against* a certain or probable sepsis for the purpose of securing asepsis. *Let us give to such work the name of the* METHOD *by which we are endeavoring to get the best results, rather than the condition we are striving for, by the use of the method.* For the benefit of those of you who have not given this subject of modern surgery much study or thought, and yet like myself are eager to get hold of any details or experiences calculated to assist us in keeping up with those younger members of the profession, fresh from the teaching of men expert in modern surgical procedures, permit me to add a few words more as how best to bring about aseptic results.

Following out this line of thought, that of securing aseptic conditions by the use of antiseptics—ANTISEPTIC SURGERY—let us look into the details necessary to be followed. First, then as to what sterilizers to use and how to use them. Heat, a temperature of 212 degrees F. is the most effective, the most surely destructive to the life of the germs, but this can be used only in sterilizing instruments or for the destruction of life in water. Water that has been boiled should, in all cases where it is possible to obtain it, be used as the menstruum in which to submerge our instruments, or for uses about the person of the operator and assistants, or around the surface about the wound. A goodly number of surgeons use hot water only, dispensing with the chemical sterilizers, such as carbolic acid, bichloride of mercury, or iodoform, either in solutions or in the dressings, yet it is but a very small proportion of such operators, who are as certain of aseptic results, as those who add to the sterilized water some one or other of the germicides. In abdominal work it is not considered safe or necesssary to use other than water sterilized by heat to the peritoneum, because of the danger of absorption, nor is it as important to do otherwise, from the fact that the solutions of continuity in this kind of work are slight and superficial in most cases, making surfaces much more easily cleaned mechanically than in deeper incisions. In a paper read before the New York Medical Association in October, 1887, Dr. Varick, of Jersey City, gave statistics collected from various sources, of 6,000 amputations, as by the old method, showing a mortality of 32 per cent. Of 53 amputations made by himself, in which he had used hot water only, he had but three deaths, a mortality of less than six per cent. He used hot water at a temperature but little below the boiling point. It was brought directly from the kettle, and he judged when it reached the wound had a temperature of about 183 degrees F. "Its effect was to hermetically seal the blood-vessels by coagulation of the albumen, thus preventing the entrance of pathogenic germs." As long as bleeding coutinues there is little danger of septic infection. "A wound that bleeds well, heals well," is an old saying.

Another advantage of hot water is, that it is a cardiac stimulant, and tends to avert the danger of shock, which is present in a greater or less degree in all major operations.

Hot water then, as used about wounds, cannot be classed as a germicide, for we are unable to put it upon living tissues at a sufficiently high temperature without destroying the wound surfaces.

For the purpose of surgical practice where the condition has no connection with micro-organisms, simple aseptic solutions, such as hot water, for the removal of blood, etc., under the head of general surgical cleanliness, is *all* that is necessary and will in very many cases produce good results. When, however, the operator must deal with a tissue which is germ infected, or which he has any reason to believe may possibly be so, he must for greater security use a germ destroying agency, that is a germicide.

My time is too limited to enter into any lengthy discussion of the various germicides. It is sufficient to mention two which are strictly germicidal, carbolic acid and corrosive sublimate; and one, iodoform, which is probably more inhibitory than germicidal in its action. These three antiseptics are the ones now used almost exclusively by modern operators, and the results obtained by their use are so very satisfactory as to appear to furnish all we need to give perfectly aseptic results.

I am well aware that several of you before me do get excellent results without the so-called germicides in your surgical work and that you feel that it is due to the care you exercise to insure cleanliness, but I am equally confident that if you should follow for a short time a surgeon who also added to his "cleanliness schedule" the use of antiseptics, you would find his results so much more certain and satisfactory that you would be persuaded to add the use of antiseptic cleanliness in your operations.

As to the first named germicide, carbolic acid, a solution of one part to twenty of water will destroy the staphylococcus aureus in 1-4 of a minute, while a solution of one to three hundred simply inhibits or suspends its function, and one of one part to three hundred and fifty will permit the micro-organism to flourish luxuriantly. The use of this germicide in surgery is confined now to the sterilization of instruments, a one to twenty solution being used for their submersion. Even when used about raw surfaces in weaker solutions, it is objectionable from the fact that what appears to be a solution in water is shown by the microscope to be simply a minute mechanical division into globules, each of which may act as a minute caustic point upon the living tissue cells.

Corrosive sublimate is the universal germicide in all operative surgery, being used for the sterilization of the person of the operator as well as of the patient, and for keeping the parts in and around the wound aseptic. But for the fact that it tarnishes instruments, it would take the place of carbolic acid as a sterilizer for them. It destroys micro-organisms in solutions of—

1 to 500 in 10 seconds.
1 to 1,000 in 45 seconds.
1 to 2,000 in 1 1-3 minutes.
1 to 4,000 in 2 1-2 minutes.
1 to 20,000 in 12 to 15 minutes.

Upon the subject of these germicides, I would recommend the reading of two valuble papers published in the MEDICAL

RECORD, one on August 3d, 1889, upon "Relative Germicidal Value of the so-called Antiseptics," by Dr. J. E. Weeks, of New York; and the other upon "The Inhibitory Action of Antiseptics," by Dr. G. W. McCaskey, of Fort Wayne, Ind., in the issue of July 13th, 1889.

And now lastly, as to that most villainous of all compounds, iodoform.

Recent investigations, notably those of Drs. Heyn and Rosing of Copenhagen, have proven that germs may live in idoform, and that in order to make it perfectly aseptic it must be itself sterilized. It is not a powerful antiseptic nor destructive to the life of micro-organisms. Dr. de Ruyter, of Berlin, however, claims it to be a most valuable antiseptic, as it limits the advance of pathological processes by reason of its destructive effect upon the ptomaines, those poisonous alkaloids produced by the decomposition of tissue cells by the action of bacteria upon them.

These ptomaines, in addition to being poisonous to the system, aid the destructive action of the bacteria upon tissue cells. Now, iodoform, while it is not claimed to seriously hinder these germs in their destructive work, does destroy the results of that work, and either enables the tissue cells to destroy them or to resist their action. In practice, iodoform is used chiefly in granulating wounds or as an antiseptic dressing.

By some operators, however, the surfaces of all wounds, whether probably aseptic or not, are dusted with iodoform before the sutures are put in, and recently the whole wound has been filled with iodoform gauze, and allowed so to remain for two or three days before coaptation is made, when union without suppuration has been secured. In small wounds nicely approximated, a 10 per cent. iodoform collodion is often used with excellent aseptic results. Bacteriological experiments are not in favor of iodoform as an antiseptic, but whatever may be urged against it theorectically, practical surgical experience continues to favor its use. Whatever may be the individual experience and opinion of any of you upon this question of antiseptics, it must be evident to all of us that cleanliness, *perfect asepsis*, is the condition in wounds desired by all operators of the present day, and must be rigidly sought for and obtained, in order to get aseptic results. The younger surgeons in our ranks, who have been educated by believers in germicides to wound surfaces, and have seen the remarkable results of such use, are almost to a unit, firm converts to the opinion that there are few strictly aseptic results without the use of antiseptics, and very many of us, who took our first lesson in surgical work prior to the era of Listerism, agree with them, that the most perfect unions are only possible when we use germicides in our solutions or dressings. Whatever may be the views you hold, it is evident to every one, that like the influence of homœpathy in moderating the drugging of the old school, so the new theory of the value of antiseptics, has so influenced us in our operative work that we are much more careful than formerly, to "clean," as perfectly as our means will allow, any wound we may produce, or that we may have to treat, already made. Those who do not use antiseptics, and yet claim aseptic results, either deny the possible presence of micro-organisms capable of producing destructive changes upon tissue cells in a wound, or they do not believe that germicides, in the strengths in which they may be safely applied to living tissues, can destroy the bacteria. How any one who has studied the investigations of bacteriologists, or has followed the results obtained by operators, using antiseptics, can deny either statement, I, from my limited study and experience, cannot conceive. The future may decide that their arguments are correct, but to us who have earnestly compared in our experiences the results of both theories, it is incredible how such an assumption will ever be sustained.

To those of you who have never used germicides in your operative work, let me make the suggestion that you apply patiently and thoroughly the principle, and with the earnest desire to solve the question in your minds, and to the others who have tried, and either from theoretical grounds or from experience, feel the principle of the theory of antiseptics is founded upon a fallacy, let me join my pleadings with those of many others, that you try again, feeling as we do, that you will, by perseverance, become, not only converts, but *enthusiasts* in the use of antiseptics applied for the purpose of obtaining perfect asepsis.

